# Methods for Screening and Isolating Extremely Heat-Resistant *Escherichia coli* from Meat Sources

**DOI:** 10.3390/life14091123

**Published:** 2024-09-05

**Authors:** Manita Guragain, Gregory E. Smith, Joseph M. Bosilevac

**Affiliations:** 1 Characterization and Interventions for Foodborne Pathogens Research Unit, Eastern Regional Research Center, Agricultural Research Services, US Department of Agriculture, Wyndmoor, PA 19038, USA; 2 Meat Safety and Quality Research Unit, US Meat Animal Research Center, Agricultural Research Services, US Department of Agriculture, Clay Center, NE 68933, USA; greg.smith@usda.gov (G.E.S.); mick.boselivac@usda.gov (J.M.B.)

**Keywords:** food safety, transmissible locus of stress tolerance, extreme heat resistance, *E. coli*

## Abstract

Meat animals harbor diverse *E. coli* populations in their digestive tracts and can serve as sources of pathogenic *E. coli*. The consumption of meat and produce contaminated with virulent *E. coli* from animal sources is associated with human illnesses and outbreaks. Heat treatment is an antimicrobial intervention that is commonly used during meat processing to ensure effective reductions in microbial load. Extreme heat resistance (XHR) has been reported among meat-borne *E. coli* and is mainly attributed to an ~15–19 kb genetic element known as the transmissible locus of stress tolerance (tLST). XHR *E. coli* can resist treatments used during meat processing and cooking. Therefore, the detection of heat-resistant *E. coli* is important for devising effective control measures to prevent meat spoilage and ensure meat safety. Here, we present methods used to (1) screen for tLST genes by multiplex PCR and (2) screen and isolate XHR *E. coli* from meat sources. The mode of heat exposure affects the outcome of XHR testing. Hence, the protocols were optimized to achieve maximum agreement between the tLST genotype and the XHR phenotype.

## 1. Introduction

*E. coli* are commensal residents of the intestinal tracts of ruminant and non-ruminant meat animals. Among the resident *E. coli* is a subpopulation that is virulent to humans. It can contaminate final animal protein products during various processing stages [1]. The meat industry routinely utilizes several antimicrobial interventions like 50 ppm sodium hypochlorite, 400 ppm bromine, 200 ppm peroxyacetic acid, 4% lactic acid, and hot water (82 °C) or steam during various stages of processing to reduce the microbial load on meat surfaces [2]. Despite these interventions, virulent *E. coli* from meat animals and meat products can make their way to consumers, leading to reports of food-borne diarrheal illness and outbreaks [3].

Extremely heat-resistant (XHR) *E. coli* from a meat processing plant were reported in 2011 [4]. Since then, XHR *E. coli* have been identified in diverse environments like meat animals and meat products [5,6,7], meat processing plants [6], raw milk cheese [8,9], and chlorinated sewage [10]. The XHR phenotype has also been identified among other bacteria like *Cronobacter sakazakii* [11], *Salmonella Senftenberg* [12], *Klebsiella pneumoniae* [13], *Pseudomonas aeruginosa* [14], and *Enterobacter cloacae* [15]. The genetic determinant of the XHR phenotype was identified as the transmissible locus of stress tolerance (tLST), a 15–19 kb locus located on the chromosome and/or a plasmid [16,17]. The tLST genes (16–19) have predicted functions against heat stress, envelope stress, and oxidative stress [18]. In addition, the tLST genes have been shown to protect against heat, chlorine, hydrogen peroxide, peroxyacetic acid, and high pressure [19,20,21,22]. Two major variants of the tLST (~61–80% nucleotide sequence identity) have been identified, tLST1 (15 kb) and tLST2 (19 kb). An individual *E. coli* isolate can carry tLST1, tLST2, or both. Both variants have similar genetic synteny and share 12 core genes. A hybrid variant of the tLST has also been identified [16,23]. Each tLST variant is associated with one of the two variants of ClpK, an ATP-dependent protease with a predicted function in heat resistance (*clpK1* is associated with the 15 kb tLST1, and *clpK_2_* is associated with the 19 kb tLST2). The ClpK variants are known to have complementary roles in heat resistance [8,24].

XHR *E. coli* can survive meat processing as well as cooking [4]. The tLST is mobile in nature, and its transfer has been shown to impart a gain of the XHR phenotype in recipient cells [19]. Resistance to heat interventions undermines meat safety and poses a public health risk. Therefore, this study aimed to develop an effective screening method for the XHR phenotype in cattle fecal samples as well as isolated bacteria.

Here, we present genetic and phenotypic methods that have been successfully employed to identify and isolate XHR *E.* coli from meat and animal sources [23,25,26]. A multiplex PCR-based method was developed to screen for intact or non-intact tLST in cattle fecal samples and bacterial isolates from various meat animal sources (beef, veal, sheep, and pork). Phenotypic assays were developed to screen XHR *E. coli* from cattle fecal and meat sources, and they were optimized to achieve maximum agreement between the tLST genotype and the XHR phenotype. Additionally, methods to isolate XHR *E. coli* from cattle feces and to differentiate the degree of heat resistance among XHR isolates are further discussed.

## 2. Materials and Methods

### 2.1. Samples and Growth Conditions

Cattle fecal samples and previously isolated *E. coli* archived at the U.S. Meat Animal Research Center were utilized for the protocols described here. The first set of samples were fecal samples (n = 1438) collected from nine processing plants in five geographically distinct regions of the United States. The fecal samples were collected from three cattle production sources (cull dairy (n = 425), fed beef (n = 538), and cull beef (n = 475)) and processed as described in [26]. The second set of samples comprised *E. coli* isolates (n = 4123) previously collected from various animal sources (beef, n = 1548 [27,28,29]; veal, n = 994 [30]; sheep, n = 511 [31]; and pork, n = 1070 [32,33]) during different meat production and processing steps (feces, n = 243; skin/hide, n = 489; preintervention carcass, n = 991; final carcass, n = 496; and finished product, n = 1094) during animal harvesting in commercial meat processing plants, as described previously. The third set of samples were generic *E. coli* (n = 232) previously isolated from three production lots of beef cattle across different stages of the beef processing continuum (feedlot feces, 36; feedlot hides, 36; harvest feces, 36; harvest hides, 36; pre-evisceration carcasses, 36; final carcasses, 28; and packaged strip loins, 24), as described in [34].

Frozen glycerol stocks of fecal enrichments were revived by subculturing the enrichments (1:10) in tryptic soy broth (TSB; Remel, Lenexa, KS, USA) with a phosphate buffer (30 gm of TSB, 2.31 g of KH_2_PO_4_, and 12.54 g of K_2_HPO_4_ per liter). *E. coli* glycerol stocks were revived by subculturing on tryptic soy agar (TSA; Beckton Dickinson, Sparks, MD, USA), and an isolated colony was subcultured in buffered peptone water (BPW; BD, Sparks, MD, USA). Primary cultures of fecal enrichments or *E. coli* isolates were further subcultured in fresh BPW (1:100) to remove the protective effects of glycerol, carbohydrates, and pH buffers. Samples were incubated at 37 °C for 16–18 h unless otherwise noted. 

### 2.2. Multiplex PCR to Screen for tLST

Multiplex PCR was designed, validated, and utilized to screen for the tLST. To detect tLST1, primers (Table 1) were designed to target the 5′ region, the 3′ region, and two internal regions of the tLST from the prototypical XHR *E. coli* AW1.7 and to differentially amplify non-overlapping regions across the tLST. Multiplex PCR was optimized to yield a four-plex assay that allowed the differentiation between intact and non-intact tLST. Nucleotide differences between *clpK1* and *clpK_2_* were exploited to identify the tLST2 variant. Primers clpK2F and clpK2R were designed to target and amplify *clpK_2_* region based on the sequence of *E. coli* strain FAM21805. (Table 1).

For PCR screening of the tLST and *clpK_2_*, each strain was grown overnight in BPW at 37 °C. For tLST detection, the PCR mix per reaction was prepared as follows: 2.6 µL of 10X buffer I containing 15 mM MgCl_2_, 0.36 mM of MgCl_2_, 0.48 µM of equimolar dNTP mix, primer pair mix (0.57 µM of 1266F/1373 R; 0.19 µM of 4295F/4500R; 0.19 µM of 7069F/7069R; 0.71 µM of 14160F/14699R), 1.5 U of Bulls eye HS-Taq polymerase (Midsci. St Louis, MO, USA) and 1 µL of the BPW overnight culture as template. The final volume was adjusted to 21 µL with DNA-free water. Step gradient thermocycling conditions were utilized for amplification: initial denaturation was at 95 °C for 5 min; followed by 10 cycles of 94 °C for 1 min, 62 °C for 2 min, and 72 °C for 1.5 min; and then 5 cycles of 94 °C for 1min, 61 °C for 2 min with 1 °C reduction in the annealing temperature per cycle, and 72 °C for 1.5 min; followed by 10 cycles of 94 °C for 1 min, 57 °C for 2 min, and 72 °C for 1.5 min; and finally, 11 cycles of 94 °C for 1min, 57 °C for 2 min, 72 °C for 1.5 with 6 s increase in the extension time per cycle. For *clpK_2_* amplification, the PCR mix for per reaction was prepared as follows: 2.6 µL of 10X buffer II containing 15 mM MgCl_2_, 0.36 mM of MgCl_2_, 0.48 µM of equimolar dNTP mix, 0.57 µM of primers ClpK2F and ClpK2R, 1.5 U of Bulls eye HS-Taq polymerase, and 1 µL of BPW overnight culture as template. Final volume was adjusted to 21 µL with DNA free water. The following thermocycling conditions were utilized for *clpK_2_* amplification: initial denaturation at 95 °C for 8 min; then, 33 cycles of 95 °C for 30 s, 57 °C for 30 s, and 72 °C for 45 s; and a final extension at 72 °C. The PCR products were held at 7 °C until further use.

Amplicons were resolved on 1.5% agarose gels by electrophoresis in 13 mM sodium borate buffer (Faster Better Media LLC, Hunt Valley, MD, USA). The amplified bands were stained with ethidium bromide (Sigma-Aldrich, St. Louis, MO, USA) and visualized under UV light using a GelDoc Go Imaging System (Bio-Rad, Hercules, CA, USA). Detection of at least one amplicon was considered tLST+, and detection of all four amplicons was considered intact tLST+.

To validate the PCR assays, tLST-negative fecal samples were diluted (1:10) in phosphate-buffered TSB followed by inoculation with 2 × 10^1^ to 2 × 10^6^ CFU of *E. coli* AW1.7. The cultures were incubated at 42 °C for 8 h. Samples of one hundred microliters of each enriched preparation were boiled for 5 min, and 1 µL was used as a template for the PCR. PCR assays were carried out as described above.

### 2.3. Aluminum Heat-Block Method for Screening and Isolation of XHR Bacteria

Fecal glycerol stocks were revived and enriched as described above. Overnight cultures (500 µL) of fecal samples in 1.1 mL thin-walled MicroTubes^TM^ (DOT Scientific, Burton, MI, USA) were thoroughly mixed, and incubated in an 80 °C aluminum heat block (Analog HeatBlock, VWR) for 15 min. The tubes were then immediately transferred and cooled in ice cold water for 45 s. Cells were allowed to recover at 22 °C for 1 h, after which an equal volume of double-strength Difco MacConkey broth (BD) was added, followed by incubation at 37 °C for 18–24 h. The growth of surviving Gram-negative bacteria was indicated by yellow coloration of MacConkey broth.

For isolation of XHR bacteria, MacConkey broth cultures positive for growth were further subcultured on Difco-sorbitol MacConkey agar containing 5-bromo-4-chloro-3-indolyl-β-D-glucuronide (BCIG-SMAC; Oxoid, Basingstone, UK) by streaking for isolation. BCIG-SAMC allowed multiple colony types to be distinguished if present in the same sample. Unique colonies were picked and transferred to 1 mL TSB and incubated at 37 °C, for 18–24 h. The isolates were tested for XHR phenotype (see below) and tLST genes as described above. XHR isolates were confirmed by yellow coloration of MacConkey broth. For identification of XHR isolates, Fluorocult LMX broth (EMD Chemicals Inc., Darmstadt, Germany), indole test and API-20 E biochemical panel (bioMérieux, Hazelwood, MO) were utilized. *E. coli* strain AW1.7 was used as a positive control for the XHR phenotype. The assay for XHR *E. coli* isolation from fecal samples was validated using spiked fecal samples as described in the previous section and compared to recovery of the reference strains AW 1.7.

### 2.4. Aluminum Heat-Block Method for Screening of XHR Phenotype in E. coli Isolates and Identification of Heat-Resistance Magnitude

*E. coli* isolates (n = 4123) previously isolated from various steps in animal harvest were utilized for this protocol. Isolate glycerol stocks were revived and enriched as described above. The overnight cultures (500 µL) in 1.1 mL thin-walled MicroTubes^TM^ were thoroughly mixed and incubated in a 60 °C aluminum heat block for 15 min. Heat-treated samples were rapidly cooled by immediate incubation in ice bath for 45 s, and then allowed to recover at room temperature for 1 h. An equal volume of double-strength MacConkey broth was added, and the samples were incubated at 37 °C for 18–24 h. Yellow coloration of media was indicative of growth and was considered an indicator of XHR. *E. coli* strain AW1.7 was used as a positive control for XHR phenotype.

Four tLST+ XHR *E. coli* isolates were selected based on the presence or absence of *clpK_2_* to further differentiate their magnitudes of heat resistance. Isolated colonies were grown in Luria Bertani broth (LB; Beckton Dickinson, Sparks, MD, USA) at 37 °C, 150 rpm for 16–18 h. Cell density was normalized to 1.6 × 10^8^ CFU/mL by measuring OD600 and adjusting with LB broth. Three milliliters of the normalized culture was transferred to a glass tube followed by incubation in 60 °C water bath for 0, 1, 5, 10, or 20 min. Heat-treated samples were immediately diluted 1:10 in BPW at room temperature. Ten-fold serial dilutions were prepared in BPW, plated on ECC petrifilm (Neogen Lansing, MI, USA), and incubated at 37 °C for 18–24 h. Colonies were counted, and the reduction in log CFU/mL of treated versus untreated samples for each time point was calculated. For each isolate, 2 or more independent experiments were carried out, each with 3 biological replicates.

### 2.5. Water-Bath Method for Screening of XHR Phenotype in E. coli Isolates

An isolated colony was taken from LB agar and used to inoculate LB broth, which was incubated at 37 °C for 7.5 h with shaking (100 rpm). The culture was then further diluted 1:16 in fresh LB broth and incubated at 37 °C, 13–14 h, 100 rpm. Cell density was normalized to ~1.2 × 10^6^ CFU/mL by measuring OD_600_ and adjusting with additions of LB broth. Nine hundred fifty microliters of the normalized culture was transferred to thin-walled plastic tubes and then incubated in a 60 °C water bath for 20 min. Normalized culture (50 µL) was used as a no-treatment control. The heat-treated sample was immediately transferred and incubated in ice water for 45 s. The cooled sample was allowed to recover at room temperature for 1h, and was then diluted 1:10 in fresh LB broth and incubated at 37 °C for 21 h with shaking (100 rpm). Optical density at 600 nm was measured to quantify bacterial growth, and the difference between absorbances of treated and non-treated samples was calculated. Log reduction of <0.5 was considered XHR phenotype. 

## 3. Results

### 3.1. Screening of Extreme Heat Resistance Using an Aluminum Heat Block

#### 3.1.1. Heat Resistance in Cattle Fecal Samples

**Figure 1:**
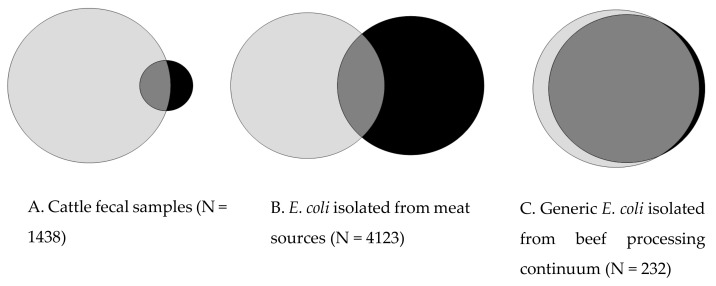
Distribution of XHR phenotype and tLST among (**A**) Cattle fecal samples, XHR measured by aluminum heat block method; (**B**) *E. coli* isolated from meat sources, XHR measured by aluminum heat block method; and (**C**) Generic *E. coli* from beef processing continuum, XHR measured by water bath method. Light grey: tLST+ only; Black: XHR only; dark grey: tLST+ and XHR. Venn diagram was generated using Venndiagramplotter 1.6.7458 (https://github.com/PNNL-Comp-Mass-Spec/Venn-Diagram-Plotter/releases, accessed on 10 July 2024).

A rapid screening method utilizing an 80 °C aluminum block was employed to screen for XHR bacteria in 1438 cattle fecal samples. The fecal enrichments in BPW were incubated in an 80 °C aluminum block for 15 min. This method identified 91 (6.3%) fecal samples containing Gram-negative XHR bacteria (Figure 1A) based on subsequent growth in MacConkey broth, as indicated by yellow coloration. Upon subculturing these 91 XHR fecal samples on the BCIG-SMAC agar, 140 isolates were recovered, and the presence of the tLST and XHR phenotype was confirmed. 

A fourplex PCR assay determined the prevalences of tLST and the intact tLST in fecal enrichments to be 59.3% (854/1438) and 18.7% (366/1438), respectively. Some disagreement between the tLST genotype and XHR phenotype was observed. The tLST was detected in only 52 out of 91 XHR samples. On the other hand, only 32 out of 854 tLST+ samples showed the XHR phenotype (Figure 1A). Further, despite 18.7% samples testing positive for an intact tLST, only 0.8% (11/1438) yielded bacteria with intact tLST. Among 140 bacteria recovered from 91 XHR positive fecal enrichments, tLST and intact tLST were detected in only 9% (13) and 7.9% (11) of isolates, respectively. A majority (125/140) of XHR isolates were identified as *E. coli,* and the remaining as *Klebsiella pneumoniae* (10/140), *Enterobacter cloacae* (4/140), and *Proteus mirabilis* (1/140). None of the tLST^+^ isolates tested positive for *clpK_2_*.

A validation assay demonstrated that the tLST was detectable in samples with pre-enrichment loads of tLST+ *E. coli* as low as 20 CFU/gm. The consistent detection of the tLST, however, required at least 2000 CFU/gm in the pre-enrichment load.

#### 3.1.2. Heat Resistance among *E. coli* Isolated from Meat Sources

The rapid screening method utilizing a 60 °C aluminum block was employed to identify the XHR phenotype among 4123 *E. coli* isolated from meat sources. An overnight BPW culture was incubated in 60 °C aluminum block for 20 min. This method identified 426 (10.3%) XHR *E. coli* based on their growth in MacConkey broth, as indicated by yellow coloration of media (Figure 1B).

The multiplex PCR assay detected tLST in 11.4% (470/4123) of *E. coli*. Similar to fecal samples, there was only 19% agreement between XHR phenotype and tLST genotype (Figure 1B). Among 426 XHR isolates, tLST was detected in 90, of which 85 showed an intact tLST. On the other hand, the XHR phenotype was not observed in 81% (381/470) of the tLST+ isolates (Figure 1B). The *clpK_2_* gene was detected in 6.5% (258/4123) of the isolates. The majority (91%) of *clpK_2_* was detected in *E. coli* with an intact tLST background, and once in an *E. coli* that lacked the tLST.

**Figure 2:**
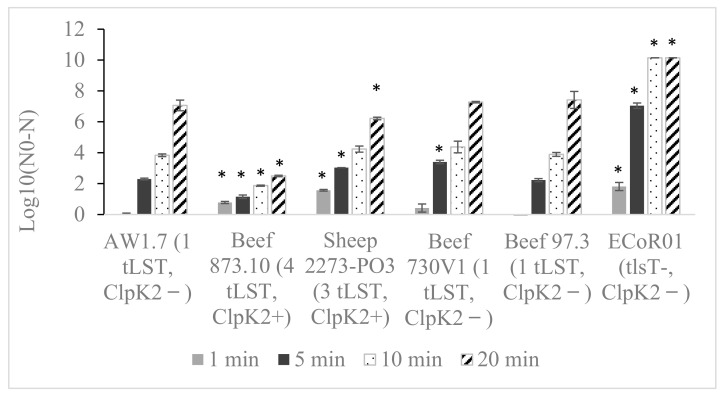
Differential heat resistance among tLST+ *E. coli* isolates from meat sources (N = 4123) as measured by refined water-bath method. Strain AW1.7 was used as a reference for heat resistance, and strain ECoR01 was used as heat sensitive control. The strains were exposed to 60 °C for 1, 5, 10, and 20 min. The data are represented as mean and standard deviation of three independent technical replicates, with each experiment independently repeated at least two times. *, *p* < 0.01 (one-way ANOVA of log CFU/mL reduction at each time point compared to AW1.7).

Previous studies have reported that the extent of heat resistance may differ between *E. coli* based on presence of one or both tLST variants [8]. Therefore, four tLST+ *E. coli* isolates with or without *clpK_2_* were selected for the refined heat-killing assay. Two isolates lacking *clpK_2_* (Beef 730V1, non-intact tLST; Beef 97.3, intact tLST) displayed heat resistance comparable to the reference XHR strain AW 1.7 (Figure 2). The reduction in Log CFU/mL observed for these strains after exposure to 60 °C for 1 min, 5 min, 10 min, and 20 min were approximately 0, 2.3, 3.8, and 7.1, respectively. The other two strains containing *clpK_2_* and an intact tLST displayed increased heat resistance compared to the reference AW1.7. Beef 873.10 was the most heat-resistant isolate, with only a 2.5 Log CFU/mL reduction after 20 min exposure to 60 °C. Strain Sheep 2273-PO3 was, however, only slightly more heat-resistant than the reference AW1.7, whereby 20 min exposure resulted in 6.2 Log CFU/mL reduction. A reference *E. coli* strain ECoR01 was used as a heat-sensitive control (reductions in Log CFU/mL of 1.8, 7.0, 10.1, and 10.1 at 1 min, 5 min, 10 min, and 20 min, respectively).

### 3.2. Heat Resistance Identified among E. coli Isolated from the Beef Processing Continuum Using the Refined 60 °C Water Bath Method

A modified rapid-screening method utilizing a 60 °C water bath was employed to screen for XHR phenotype among 232 generic *E. coli* isolated from the meat processing continuum. The overnight cultures were incubated in a 60 °C water bath for 20 min. This method identified 30 (12.9%) XHR *E. coli* based on a log reduction of <0.5 in LB broth (Figure 1C).

The multiplex PCR assay detected tLST in 14.7% (34) of the generic *E. coli*. The *clpK_2_* variant was identified in 10.3% (24) of the isolates. Among the generic *E. coli*, a high agreement between the XHR phenotype and tLST genotype was observed compared to *E. coli* sourced from pathogen focused isolations. The tLST was detected in 97% (29/30) of XHR generic *E. coli*. Among 34 tLST+ *E. coli*, the XHR phenotype was observed in 86% (29/34) of the isolates (Figure 1C). Intact tLST was detected in only 8 out of 30 XHR *E. coli*. Five tLST+ *E. coli* failed to show an XHR phenotype.

## 4. Discussion

Heat is an important intervention used in the meat industry to control pathogenic and spoilage bacteria. Extreme heat-resistant *E. coli* and its genetic marker, the tLST, are prevalent in various meat animals and in meat at all stages of processing. The detection of heat-resistant *E. coli* is important in controlling the contamination of final products by pathogenic and spoilage bacteria. A similar level of heat resistance has been reported between Shiga toxin-producing *E. coli* (STEC) and generic *E. coli* [6]; however, tLST is almost always excluded from genomes of diarrheagenic *E. coli* carrying toxin gene (*stx*) [16,17,35]. Nonetheless, the emergence of tLST-mediated XHR diarrheagenic *E. coli* or XHR spoilage bacteria cannot be ignored given the mobile nature of the tLST [8,15]. Moreover, recent studies have detected tLST in Extraintestinal pathogenic *E*. *coli* (ExPEC) and reported their reduced sensitivity to UV-C treatment [36].

The screening of heat resistance by an aluminum block method was originally developed and utilized for a rapid screening of XHR *E. coli* in fecal samples, and later used on *E. coli* isolates as well. The simplicity of the protocol combined with manual reading of MacConkey broth color provided a simple tool to screen high numbers of samples for XHR bacteria. However, the disagreement observed between the presence of tLST and the XHR phenotype indicated the need for further optimization of the protocol. Such disagreement, however, may also arise from biology of the organism. Lack of the XHR phenotype in tLST+ samples may indicate a need for an optimal genetic background for the tLST function. On the other hand, the XHR phenotype in the absence of the tLST can be indicative of novel heat-resistance mechanisms. Nevertheless, the possibility of additional stress to heat-injured cells due to use of selective media like MacConkey broth and ECC petrifilm cannot be ignored. Furthermore, others [37] have noted that slow and non-uniform heating from a dry bath can affect the outcome of XHR screening assays. Therefore, the XHR screening protocol was optimized for use with a water bath for heat treatment. The water-bath method of XHR screening showed 97% agreement between XHR phenotype and tLST genotype. Further optimization included replacement of MacConkey with non-selective LB broth to avoid additional stress on recovering cells. These refinements, together with final readings of OD_600_ in a 96-well format, made the water-bath method a reliable and high-throughput XHR-screening method. Additionally, the selected heat-treated samples can be plated on non-selective media for a more quantitative assay.

The amplification of all targets upon PCR was interpreted as an intact tLST. However, using the number of amplicons as a sole predictor of heat resistance can be misleading. An in silico tLST PCR of an XHR isolate Beef 730V1 [23] yielded all four amplicons. In contrast, only three PCR products were detected with the multiplex PCR. Further, low recovery of intact tLST bacteria from the intact tLST positive fecal samples, possibly due to a mixture of isolates providing the tLST template, makes this a less ideal approach for samples harboring mixed bacterial populations. While the molecular detection of tLST is important in estimating the potential XHR prevalence, the detection of genotype does not always result in the predicted phenotypic characteristics. On the other hand, relying on one genetic marker (e.g., tLST) can prevent the identification of novel mechanisms of heat resistance. Therefore, combining both genotypic and phenotypic approaches in a reliable and high-throughput manner is important for effective screening.

The refined assay developed for differentiation of the magnitude of heat resistance was successful in demonstrating that isolates carrying *clpK_2_* as well as more copies of the tLST display greater heat resistance. The refined assay also demonstrated that none of the isolates with less than three tLST amplicons in multiplex PCR showed XHR phenotype. The design of the assay provides more resolution needed for calculation of D-value but does not make it suitable for high-throughput screening.

## 5. Conclusions

The multiplex PCR-based assay can successfully detect tLST, a genetic marker of extreme heat resistance, in both fecal samples and *E. coli* isolates. However, caution should be exercised when interpreting intact vs. non-intact tLST, particularly in samples with mixed bacterial populations. Compared to dry heat bath, a water-bath-based method for identification of XHR phenotype yields better agreement with tLST gene, and is hence more reliable for screening of the XHR phenotype. A combination of both these genetic and phenotypic assays provides a reliable and high-throughput approach for identifying stress-resistant *E. coli* in meat.

## Figures and Tables

**Table 1:** Primer sequences used for tLST screening.

Primer	Sequence	Amplicon Size (bp)	Reference
1266 F 1372 R ^a^	5′-AATGCAGGCGGTGATGAAGA-3′ 5′-CGCTGATTGCCCATCAACAG-3′	107	[26]
4295 F 4505 R ^a^	5′-CGAGGGAGAATTCCAGTCCG-3′ 5′-GGCACTACGCTAATCCTGCT-3′	210	[26]
7069 F 7404 R ^a^	5′-CTCATTGGATGCTTCGCTGC-3′ 5′-ACGGAAACCATTGAGGCGAT-3′	335	[26]
14160 F 14699 R ^a^	5′-CCTGGCATTGTTTTCTGGCC-3′ 5′-GGCTGTTCGATGACGCATTC-3′	539	[26]
ClpK2F ClpK2R	5′-ACGATCACTATCGCCAACTG-3′ 5′-AGTATTTATCCAGCTCGGGCGTG-3′	711	[26]

^a^ Primer names designate the location of primer within the tLST sequence (National Center for Biotechnology Information accession number LDYJ01000141.1).

## Data Availability

The data presented in this study are available in Science Direct at DOI 10.4315/JFP-20-103 [26] and DOI 10.1016/j.jfp.2022.100031 [25], and in PubMed Central at DOI 10.1128/AEM.02343-20 [23].
